# Can integrated health services delivery have an impact on hypertension management? A cross-sectional study in two cities of China

**DOI:** 10.1186/s12939-016-0485-7

**Published:** 2016-11-30

**Authors:** Haitao Li, Ying Sun, Dongfu Qian

**Affiliations:** 1 School of Medicine, Shenzhen University, Shenzhen, China; 2 The Affiliated Hospital of Qingdao University, Qingdao, China; 3 School of Health Policy and Management, Nanjing Medical University, Hanzhong Road 140, Nanjing, 210029 Jiangsu Province China

**Keywords:** Primary care, Continuity of care, Hypertension, High blood pressure

## Abstract

**Background:**

Policy makers require information regarding performance of different primary care delivery models in managing hypertension, which can be helpful for better hypertension management. This study aims to compare continuity of care among hypertensive patients between Direct Management (DM) Model of community health centers (CHCs) in Wuhan and Loose Collaboration (LC) Model in Nanjing.

**Methods:**

A cross-sectional questionnaire survey was conducted. Four CHCs in each city were randomly selected as study settings. 386 patients in Nanjing and 396 in Wuhan completed face-to-face interview surveys and were included in the final analysis. The relational continuity and coordination continuity (including both information continuity and management continuity) were measured and analyzed. Binary or multinomial logistic regression models were used for comparison between the two cities.

**Results:**

Participants from Nanjing had better relational continuity with primary care providers as compared with those from Wuhan, including more likely to be familiar with a CHC physician (OR = 2.762; 95%CI: 1.878 to 4.061), taken care of by the same CHC physician (OR = 1.846; 95%CI: 1.262 to 2.700), and known well by a CHC physician (OR = 1.762; 95%CI: 1.206 to 2.572). Multinomial logistic regression analyses showed there were significant differences between the two cities in reported frequency of communications between hospital and CHC physicians (*P* = 0.001), whether hospital and CHC physicians gave same treatment suggestions (*P* = 0.016), as well as how treatment strategy was formulated (*P* < 0.001). Participants in Wuhan were less likely than those in Nanjing to consider there was continuum regarding health services provided by hospital and CHC physicians (OR = 3.932; 95%CI: 2.394 to 6.459).

**Conclusions:**

Our study shows that continuity of care is better for LC Model in Nanjing than DM Model in Wuhan. Our study suggests there is room for improvement regarding relational and information continuity in both cities.

## Background

Hypertension is an important public health problem faced by policy makers worldwide and China. It is reported that one in three adults had hypertension throughout the world in 2012 [1]. It is projected that there will be 1.56 billion adults living with hypertension in 2025 [2]. In China, prevalence of hypertension has increased sharply during the past several decades – escalated from about 8% in 1979 [3] to 34% in 2010 [4]. World Health Organization (WHO) recognizes hypertension as one of the most important causes of premature death. Estimates suggest that hypertension causes about 7.5 million deaths annually, accounting for 12.8% of total deaths in the world [5]. In China, about 50% of deaths are attributable to pre-hypertension and hypertension, which is recognized to be the leading cause of premature death [6].

Hypertension control is thus important to reduce the risks of diseases with hypertension as a key risk factor. However, hypertension is sub-optimally controlled throughout the world including China. A study with a nationally representative sample of US population showed that hypertension control rate was 53% in 2010 [7]. In Canada, approximately 66% of hypertensive patients had their blood pressure under control [8]. The Chinese national hypertension survey in 2002 showed that only 4% of patients with hypertension met blood pressure control target [9]. The WHO Report 2008 has emphasized the relevance of primary care in coping with the increased challenges of chronic diseases including hypertension. The New Healthcare Reform Plan launched by the Chinese government in 2009 highlighted the key role of primary care in managing hypertension [10]. The WHO Report 2013 outlines primary care programs for any country initiative to address hypertension.

In China, primary care, named as community health services, is usually provided by community health centers (CHCs) in urban areas and township hospitals in rural areas. Six-integrated health services are designed to be provided by primary care facilities including medical care, preventive care, rehabilitation, health education and promotion, chronic disease management and technical support for family planning [11]. Health personnel usually consist of general practitioners, Traditional Chinese Medicine doctors, nurses and public health doctors etc [12]. Usually, there is a loose collaboration between hospitals and CHCs, namely Loose Collaboration Model (LC Model). CHCs in Nanjing City, Jiangsu Province are LC Models, which are separate and independent organizations from hospitals. Hospitals just provide technical guidance to CHCs, such as training sessions on specific topics. International experience has shown that close cooperation between hospitals and primary care facilities can provide patients with a seamless and interconnected healthcare, which is widely recognized to be able to improve quality of care and lower healthcare costs [13, 14]. Primary care reform in China has targeted increased service integration and multidisciplinary coordination. One model of care for integrated health services delivery has emerged, i.e., Direct Management Model (DM Model). Wuhan City, Hubei Province is a pioneer in exploring and developing the DM Model in China. In this model, CHCs are similar to departments of a hospital. Hospital is responsible for the overall operation of its affiliated CHCs, playing an important financial and administrative role. For example, the personnel of CHCs are managed by the hospital.

Continuity of care is defined by the Institute of Medicine as one key attribute of primary care [15]. Continuity of care refers to both relational continuity between patient and primary care provider and care continuity between primary care provider and specialist such that a patient seamlessly experiences care across different providers (coordination) [16]. The longitudinal relationship ideally leads to a bond between physicians and patients, characterized by trust and a sense of responsibility [17]. Coordination continuity consists of information continuity which refers to communicating timely clinical and life information about a patient’s condition between providers, and management continuity which means coordinating medical services and care pathways between multiple providers and settings in response to ongoing care needs [18]. Continuity is essential for the care necessary for chronically ill patients, since cultivating a relationship with a single provider builds up knowledge of a patient’s preferences and can help integrate a patient’s care between different providers.

Primary care reform in China has targeted increased service integration and multidisciplinary coordination. DM Model is the latest form of CHC-hospital collaboration. Policy makers require information regarding performance of DM Model in managing hypertension, which can be helpful for better management of hypertension and in ensuring better development of DM Model. To our knowledge, the information is scarce with respect to performance of DM Model in managing hypertension. This study aimed to compare performance of DM Model in Wuhan with LC Model in Nanjing in managing hypertension, which is measured by continuity of care.

## Methods

### Participants and procedures

A cross-sectional study was conducted in Wuhan and Nanjing in 2012. Nanjing is the capital of Jiangsu Province and the second largest city in eastern China, while Wuhan is the capital of Hubei Province and the most populous city in central China. Both Nanjing and Wuhan hold sub-provincial administrative status. In 2013, GDP per capita was RMB 98 thousand (US$16 thousand) for Nanjing [19], and RMB 89 thousand (US$14 thousand) for Wuhan [20]. Both cities are leading primary care development in their own CHC-hospital collaboration models. A multistage sampling method was used to select CHCs as study settings. In the first stage, one typical tertiary general hospital that has developed cooperation relations with local CHCs was selected in each city using a representative sampling. In the second stage, deployed simple random sampling methods, four CHCs in each city, which collaborated with the recruited typical hospitals, were selected randomly. At last, a total of 8 CHCs were selected as study settings. In this study, all selected CHCs and hospitals are public health institutions and owned by the government.

The sampling frame was CHC users’ hypertensive population based. It was shown that a maximum sample size of 300 per group was needed to generate a 95% confidence interval and 90% statistical power [21]. Inclusion criteria of participants were: 1) aged between 18 and 80 years; 2) with confirmed diagnosis of hypertension; 3) living in the catchment area of CHC ≥1 year; 4) had experience of both specialty care and primary care utilization during the past 1 year period. We excluded patients who had severe hypertension complications and terminal illnesses. A systematic sampling design was adopted to ensure that only patients coming to the CHC for primary care during the survey period were invited for inclusion. A hundred eligible patients were approached in each included CHC. Extensively trained interviewers performed on-site based face-to-face interview surveys. Participants were assured of anonymity and confidentiality of the survey, and verbal informed consent was obtained before the surveys were commenced. Among the approached patients, 386 in Nanjing and 396 in Wuhan completed face-to-face interview surveys with a response rate of 96.5 and 99.0% respectively. The study was approved by the Research Ethics Committee of Nanjing Medical University.

### Key measures

We conceptualized three dimensions of continuity of care (i.e., relational continuity, information continuity and management continuity) in Haggerty’s model of continuity of care. To understand the longitudinal relationship between primary care provider and patient (relational continuity), we asked three questions. 1) Is there any CHC physician that you are familiar with? 2) When you go to CHC, are you taken care of by the same physician each time? 3) Does the physician in CHC knows you well, including your most important health problems, your complete medical history, and medications you are taken?

As for coordination continuity, we asked several questions to measure information continuity. 1) How often do hospital-based physicians communicate with primary care providers (CHC physicians)? 2) How do you think the communication results between hospital-based physicians and primary care providers (CHC physicians)? 3) Does your specialist give the same treatment suggestions as that of your primary care providers (CHC physicians)? 4) Who formulated treatment strategy for you? As for management continuity, it was measured by one question: 1) Do you think there is continuum regarding health services provided by hospital-based physicians and primary care providers (CHC physicians)?

According to the framework of Behavioral Model of Health Services Utilization, individual factors that may influence health care use were collected. Individual factors consist of predisposing factors (including gender, age, marital status and education level), enabling factors (including monthly household income), as well as need factors (including year of hypertension since diagnosis, complications, self-reported overall health status and blood pressure measures). We grouped marital status into two categories, those single (including not married, separated, widowed and divorced), and those currently married or cohabited. Education level was collapsed into four categories, i.e., primary school and below, middle school, high school and equivalent, as well as 3-year college and above. According to mean monthly household income in 2012 (RMB3000 for Nanjing [19], and RMB2400 for Wuhan [20]), the participants were grouped into two economic levels– below or above mean monthly household income. Hypertension-related complications included cerebrovascular disease, heart disease and kidney disease. Blood pressure (BP) was measured by trained interviewers according to Chinese Hypertension Management Guidelines. BP control was determined by whether a participant met BP control target level, i.e., <140/90 mmHg.

### Statistical analysis

The socio-demographic characteristics and hypertension-related information between the participants from the two cities were compared by using Chi-square tests (or independent two-sample t-tests where appropriate). We firstly employed Chi-square tests to compare participants’ reported experiences of continuity of care between the two cities. Then, binary or multinomial logistic regression models were used for comparison between the two cities by controlling for participants’ gender, age, education level, marital status, monthly household income, overall health status, year of hypertension since diagnosis and hypertension-related complications. All data were tested to establish if they violated assumptions of the multinomial logistic regression; though this type of regression does not assume normality, linearity, or homoscedasticity. Adjusted odds ratio (OR) with 95% confidence interval (CI) was reported where Wuhan was regarded as the reference group. The likelihood ratio test statistic was used to test the fit of model. For all tests conducted in the study, a *P* value of less than 0.05 was adopted as the statistically significant level. All analyses were performed by using SPSS19.0.

## Results

Compared with those from Wuhan, participants from Nanjing were more likely to be male (51.3 vs 43.2%, *P* = 0.026). In both cities, the majority of participants tended to be aged more than 60 years old (69.8 vs 87.4%) and married or cohabited (91.2 vs 89.4%). Participants from Nanjing tended to possess a higher education level when compared with those from Wuhan (*P* < 0.001). More participants’ monthly household income in Nanjing than in Wuhan was higher than the mean level in each city (*P* < 0.001), while more than one third participants from Nanjing rejected to report their income. Participants’ overall health status in Nanjing was poorer than those in Wuhan (*P* = 0.009). When compared with those from Wuhan, participants from Nanjing were less likely to have hypertension-related complications (32.8 vs 48.3%, *P* < 0.001), while more likely to have their BP under control (38.9 vs 28.9%, *P* = 0.003) (Table 1).

**Table 1:** Characteristics of the participants by city

Characteristics	Nanjing N(%)	Wuhan N(%)	*P* value
*Socio-demographic*			
Gender			0.026
Male	198(51.3)	171(43.2)	
Female	188(48.7)	225(56.8)	
Age			<0.001
< 60	118(30.2)	50(12.6)	
> =60	273(69.8)	348(87.4)	
Marital status			0.464
Single	33(8.8)	41(10.6)	
Married or cohabited	340(91.2)	347(89.4)	
Education			<0.001
Primary school and below	71(18.7)	123(31.1)	
Middle school	95(25.1)	148(37.4)	
High school and equivalent	107(28.2)	87(22.0)	
3-year college and above	106(28.0)	38(9.6)	
Monthly household income			<0.001
Low	45(11.5)	110(27.6)	
High	195(49.9)	274(68.8)	
Rejected	151(38.6)	14(3.5)	
*Disease-related*			
Year of hypertension (mean, SE)	10.48(0.51)	11.91(0.50)	0.046
Health status			0.009
Good	153(40.2)	197(49.6)	
Fair or poor	228(59.8)	200(50.4)	
Complications			<0.001
Yes	124(32.8)	185(48.3)	
No	254(67.2)	198(51.7)	
BP control			0.003
Yes	152(38.9)	115(28.9)	
No	239(61.1)	283(71.1)	

*SE* standard error

Significant differences between the two cities were identified regarding relational continuity. After adjusting for socio-demographic characteristics and hypertension-related factors, participants from Nanjing tended to report that there was a CHC physician that they were familiar with (56.4% vs 31.5%; OR = 2.762; 95%CI: 1.878 to 4.061); they were taken care of by the same CHC physician each time (47.1% vs 33.4%; OR = 1.846; 95%CI: 1.262 to 2.700); and, CHC physician knew them well including their most important problems and complete medical history (58.3% vs 46.8%; OR = 1.762; 95%CI: 1.206 to 2.572) when compared with those from Wuhan (Table 2).

**Table 2:** Patient reported experiences of continuity of care by city

Variables	Nanjing	Wuhan	*P* value^a^	OR (95% CI)^b^
*Relational continuity*				
CHC Physician you are familiar with			<0.001	2.762(1.878,4.061)***
Yes	212(56.4)	123(31.5)		
No	164(43.6)	267(68.5)		
Same CHC physician every time			<0.001	1.846(1.262,2.700)**
Yes	179(47.1)	132(33.4)		
No	201(52.9)	263(66.6)		
CHC physician knowing your disease history			0.002	1.762(1.206,2.572)**
Yes	214(58.3)	182(46.8)		
No	153(41.7)	207(53.2)		
*Information continuity*				
Frequency of communications between CHC and hospital physicians			<0.001	
Many	26(7.0)	18(4.8)		1.460(0.637,1.024)
Fair	71(19.1)	33(8.7)		3.019(1.723,5.288)***
Few	70(18.9)	58(15.3)		1.695(1.020,2.817)*
Do not know	204(55.0)	269(71.2)		Ref
Communication results			<0.001	
Good	33(10.2)	10(3.6)		2.173(0.848,5.567)
Fair	209(64.1)	119(53.1)		1.664(1.019,2.718)**
Poor	84(25.8)	95(42.4)		Ref
Whether hospital and CHC physicians have same suggestions			0.001	
Yes	119(33.1)	83(23.0)		1.869(1.211,2.884)**
No	26(7.2)	15(4.2)		1.543(0.666,3.578)
Do not know	215(59.7)	263(72.9)		Ref
How your treatment strategy was formulated			<0.001	
CHC physician	190(50.5)	126(32.5)		8.553(4.247,17.229)***
Hospital physician	138(36.7)	157(40.5)		4.495(2.243,9.009)***
CHC physician together with hospital physician	33(8.8)	4(1.0)		32.825(8.355,128.963)***
No fixed strategy	15(4.0)	101(26.0)		Ref.
*Management continuity*				
Total continuum between hospital- and CHC- physician provided services			<0.001	3.932(2.394,6.459)***
Yes	232(72.7)	78(41.5)		
No	87(27.3)	110(58.5)		

*OR* odds ratio, *CHC* community health center, *Ref* reference group

^a^Chi-square tests; ^b^Binary or multinomial logistic regression models where appropriate; *P < 0.05; **P < 0.01; ***P < 0.001

As for information continuity, multinomial logistic regression analyses showed that there were significant differences between the two cities in reported frequency of communications between hospital and CHC physicians, whether hospital and CHC physicians gave same treatment suggestions, as well as how treatment strategy was formulated (Table 3). Compared with those in Wuhan, participants in Nanjing tended to report that communications between hospital and CHC physicians were fair (19.1 vs 8.7%; OR = 3.019; 95%CI: 1.723 to 5.288) or few (18.9 vs 15.3%; OR = 1.695; 95%CI: 1.020 to 2.817). More participants in Nanjing than in Wuhan reported that suggestions provided by hospital physicians were the same as that of CHC physicians (33.1 vs 23.0%; OR = 1.869; 95%CI: 1.211 to 2.884). Participants in Wuhan were less likely to report that their treatment strategy was formulated by CHC physicians (50.5 vs 32.5%; OR = 8.553; 95%CI: 4.247 to 17.229) or by CHC and hospital physicians together (8.8 vs 1.0%; OR = 32.825; 95%CI: 8.355 to 128.963) when compared with those in Nanjing (Table 2).

**Table 3:** Results from multinomial logistic regression likelihood ratio tests showing the differences between Nanjing and Wuhan

Dependent variables	Model fitting criteria	Likelihood ratio tests		
		Chi-square	df	Sig.
Frequency of communications between CHC and hospital physicians	1243.14	16.746	3	0.001
Communication results	765.501	5.206	2	0.074
Whether hospital and CHC physicians have same suggestions	924.948	8.313	2	0.016
How your treatment strategy was formulated	1353.31	56.469	3	<0.001

*CHC* community health center, *Sig* significance

When it comes to management continuity, Table 2 shows that participants in Wuhan were less likely than those in Nanjing to consider there was continuum regarding health services provided by hospital and CHC physicians (72.7% vs 41.5%; OR = 3.932; 95%CI: 2.394 to 6.459), after adjusting for participant socio-demographic characteristics and hypertension-related factors by using binary logistic regression models (Table 2).

## Discussion

Our study found that participants in Nanjing were more likely to have a CHC physician they were familiar with, be taken care of by the same CHC physician for each CHC visit, and have a CHC physician knowing their disease history. Although there were more participants in Wuhan than in Nanjing did not know whether there was any communication between hospital and CHC physicians, the participants in Nanjing tended to consider that the communications between hospital and CHC physicians were fair or few. Similarly, more participants in Nanjing than in Wuhan perceived that the suggestions between hospital and CHC physicians were same; though more participants in Wuhan did not know whether they had same suggestions. There were more participants reported that their treatment strategy was formulated by CHC physicians together with hospital physicians, although the percentages were low for both cities. In general, management continuity was perceived better by the participants in Nanjing than those in Wuhan.

Limitations of the study should be addressed. Firstly, general applicability of study findings may be limited. For one thing, the study findings cannot be extended to other cities with similar CHC models as structure of CHCs with same models may be different in different cities; for another, information were reported by CHC users and cannot be generalized to hypertensive population in general. Secondly, the representativeness of this study is limited. The representative sampling methods may introduce sampling bias. Moreover, the participants were not selected by using strict random sampling methods. Thirdly, patient-reported information may be subject to recall bias. Fourthly, the items used to measure continuity of care in our study may introduce bias. Further studies are warranted to compare continuity of care between LC Models and DM Models comprehensively. Last but not the least, cross-sectional nature of the current study warrants further investigations to establish causal inferences.

Results showed that participants in Nanjing had better experiences of relational continuity of primary care when compared with those in Wuhan. The differences in health insurance schemes between the two cities may contribute to the differences in relational continuity reported by the participants in the two cities. In Nanjing, reimbursement rate of charges for CHC services is about 10% higher than that for hospital services, while in Wuhan that difference is only about 5% which is lower than that of Nanjing. Financially, CHC provided primary care may be more attractive in Nanjing than in Wuhan. In other words, patients in Nanjing are more likely to seek primary care than those in Wuhan. Therefore, the participants in Nanjing were more likely to have a CHC physician they were familiar with. The implementation of First Contact Care Scheme may help to explain this observation too. In Nanjing, it is necessary for patients covered by the Basic Medical Insurance for Urban Residents to obtain referrals from their CHC physicians for reimbursement of charges of hospital services. The gate-keeping role of CHC physicians may lead to their knowing more about patients’ complete medical history and important health problems. Another possible explanation of this observation is the number of CHC physicians. Previous studies have shown that smaller practices had better relational continuity [22, 23]. Our study showed that average number of CHC physicians in Nanjing was smaller than that in Wuhan, which suggests that CHC physicians in Wuhan have greater sharing of clinical duties resulting in a loss of continuity. Both cities should strengthen relational continuity although it was better in Nanjing than in Wuhan. Enhanced relational continuity can improve hypertensive patients’ compliance with medications and follow-up appointments [24].

Results showed that the information continuity was poor in both Wuhan and Nanjing. It was found that the majority of participants in Wuhan did not know the communications between CHC- and hospital- physicians, while the communications between CHC- and hospital- physicians were perceived few and poor by the participants in Nanjing although the awareness rate was higher than that in Wuhan. Another item in our study also showed that less than 10% of treatment strategies were formulated by CHC- and hospital- physicians together. The poor information sharing system between CHCs and hospitals may be one possible explanation of this finding. A study by Yang et al. indicated that information sharing system between CHCs and hospitals in Wuhan has not been well established [25]. It was shown that there was a fragmentation of information systems between CHCs and hospitals in Nanjing [26]. The information sharing system can assist with access to hypertensive patients’ medical records leading to improved recognition of hypertensive patients’ problems and therapies [27], which are important for better hypertension management. Strategies like adoption of reciprocal referral system, a shift from fee-for-service to global payment, performance-based payment for care providers, and integrated information system may work together to enhance not only relational continuity but also information continuity of care to address the needs of hypertensive patients [28, 29].

This study is the first to compare performance of LC Model and DM Model in managing hypertension as measured by continuity of care. This study adds to the evidence suggesting that continuity of care is better for LC Model than for DM Model. Current literature showed mixed views regarding relationship between CHC models and quality of care. Both CHCs of LC Models and DM Models are publicly owned, but CHCs of DM Models are managed and operated by collaborated hospitals. These hospitals have to rely on profit-generating services to survive financially which influence their managed CHCs’ operation or development mode and lead to more profit-driven nature. Publicly owned CHCs are shown to provide a higher quality of care because they have stronger and better policy implementation [30], such as First Contact Care Scheme and dual-referral system. However, some other studies showed that quality of care offered by for-profit primary care facilities was better than publicly owned ones [31, 32]. The conflicting findings among different studies suggest that performance of CHCs depends more on the process of hypertension management rather than CHC-hospital collaboration models [11].

## Conclusions

Our study found that hypertensive patients in Nanjing had better relational continuity with CHC physicians than those in Wuhan. Information continuity was found poor in both Nanjing and Wuhan. Our study suggests that there is room for improvement regarding relational continuity and information continuity in both cities.

## Abbreviations

BP: Blood pressure
CHCs: Community health centers
CI: Confidence interval
DM Model: Direct management model
LC Model: Loose collaboration model
OR: Odds ratio
RMB: Ren Min Bi
WHO: World Health Organization
